# Medical Directions for a Toilette in the Seventeenth Century

**Published:** 1905-02-04

**Authors:** 


### Medical Directions for a Toilette in the Seventeenth Century.

Dr. Toby Venner, the son of a gentleman in Somerset, was born at Petherton, near Bridgwater, in 1577, and was matriculated from St. Alban's Hall, Oxford, on May 15th, 1595. He graduated B.A. February 1st, 1598–9; M.A., July 7th, 1603, and was admitted M.B. on March 10th, and M.D. on March 12th, 1612–3. He defended for his theses the propositions: (1) Whether medicine can be legally practised by those who are mere licentiates? (2) Whether a patient should be purged at the beginning of a fit of the gout? (3) Whether blood should be let in putrid fevers if there be no plethora? The first two propositions he affirmed, the last he denied. Dr. Venner practised first at Bridgwater, afterwards at Bath, where he died, March 27th, 1660, and was buried in St. Peter's Church. He was married and had several children, one son being a scholar of Wadham in 1622 and another matriculated at Trinity College at the early age of 14, in 1624.

Dr. Venner wrote the "Via Recta ad Vitam Longam; or a treatise where in the right way and best manner of living for attaining to a long and healthfull life is clearly demonstrated and punctually applied to every age and constitution of body . . . whereunto is annexed by the same author A very necessary and compendious treatise of the famous baths of Bathe with a censure of the medicinall faculties of the water of St. Vincents-Rocks near the City of Bristoll as also An accurate treatise concerning Tobacco which very many in these days do too licentiously use." The "Via Recta" was published in two parts, in 1620 and 1623 respectively. It sold well and was re-issued in 1628, 1638, and 1650. It is a popular work, somewhat on the lines of the Regimen Sanitatis Salerni, telling of the virtues and dangers of various kinds of meat and drink. The following chapter gives a fair sample of Dr. Venner's style and of the information he offers:—

"Whether drying and warming of the bed-Pelve ignito (with a warming pan) a little before entering therinto, be expedient and necessary? and what is to be done after the sleep for the health of the body before we betake ourselves to our ordinary and necessary business? I add these two queries as an appendix to this section. To the former I answer that for the aged and all such as are weak by nature and that lead a tender and delicate course of life, the custom of warming the bed is for two reasons very expedient and necessary in the cold and moist seasons of the year. The first is that the body upon putting off the garments may not on a sudden be affected with the external cold. The second is because the interior heat is comforted by the external, the concoction (digestion) holpen and all superfluous moisture the better consumed. But I approve not this custom to such as are healthful and strong because it will debilitate their bodies and make them over-nice and effeminate. It remaineth therefore that this is only convenient for the aged and all such as are weak and tender by nature.

To the second I answer, that after you have taken sufficient and competent rest, it is good before you arise out of your bed that you gently rub and stroke downward your breasts and sides, but your neck, shoulders, back, arms, hand-wrists, pinbones, thighs, and legs more strongly with your own hand, or with an hot linen cloth, doubled and heated for the purpose, or cause them to be rubbed, because it quickeneth the blood and strengtheneth the parts by exciting the natural heat. When you have risen, and before also, extend and stretch out your arms, legs, and whole body, that the animal spirits may be dilated to the exterior parts, and the limbs by that means corroborated; then walk a little up and down, that the superfluities which shall be in the stomach and other parts may the more speedily descend and be avoided, and be very diligent to excrete the urine and depose the excrements of the belly, and let not with less diligence the superfluities of the nose by exsufflation, and of the breast by expectoration, be purged forthwith, for nothing is more hurtful to the body than the retention of the excrements. That done, wash and plunge your eyes in cold water, for that not only cleanseth away the filth, but also cleareth and preserveth the sight, for outward cold strengthens the sight, but heat weakens it. And let the mouth be well cleansed with cold water, and the teeth rubbed thereupon with a coarse dry cloth. It will be the better if a little vinegar or white wine be sometimes added to the water, and the gums and teeth rubbed with a sage leaf or two dipped therein, or washed and cleansed with the effusion aforesaid, and the teeth afterwards rubbed with a coarse, dry cloth, for this purifieth the breath and preserveth the teeth from corruption. Then let your head be well combed that the pores may be opened, to avoid such vapours as yet by sleep are not consumed. And in the cold and moist seasons of the year let the head also be well rubbed with a coarse linen cloth, somewhat heated, for thereby the natural heat is excited, the pores opened, vaporous and rheumatic superfluities discussed and difflated, and consequently, the brain and animal spirits exceedingly comforted. Of this, therefore, as well as also of rubbing the neck in like manner, I wish students and all such as are subject to rheums, palsies, and such like affections of the sinews to have a special care.

And here I advise them that are subject to the Stone or Gravel in their kidneys that they weare their clothes thin and loose upon the reins of the back: but in cold seasons, especially as they grow in years, that they have some soft woollen cloth or fur, circumligated their loins, which will preserve from lumbaginous pains.

All which being done for the body, let not your better part pass neglected, but before you betake yourself to your study or such business as your place shall require, consecrate half an hour at the least unto Almighty God by pouring forth your thankful soul unto him. . . . Let the remembrance of your sins be bitter unto you: and cast not away your souls by fashioning yourself after this Pharisaicall and most sinful time (1650, two years after the martyrdom of K. Charles I.), but be holy, upright, uncorrupt, merciful, peacable: to shut up all in a word, labour by all means, to have always a clear conscience towards God and towards man."

